# Decoding the crosstalk: interplay between macrophage programmed cell death networks and cholesterol efflux

**DOI:** 10.3389/fcvm.2026.1895990

**Published:** 2026-08-17

**Authors:** Yanan Bian, Xiaoyang Hu

**Affiliations:** Basic Medical College, Heilongjiang University of Chinese Medicine, Harbin, China

**Keywords:** ABCA1, atherosclerosis, cell death bifurcation, lipophagy, macrophage programmed cell death, reverse cholesterol transport

## Abstract

Atherosclerosis is driven by the interplay between lipid accumulation, chronic inflammation, and macrophage dysfunction. Although intensive lipid-lowering therapy reduces cardiovascular risk, substantial residual risk persists, highlighting the importance of impaired macrophage cholesterol handling and reverse cholesterol transport (RCT). This review summarizes the bidirectional relationships between macrophage programmed cell death and cholesterol metabolism, with a focus on apoptosis, pyroptosis, necroptosis, and ferroptosis, which have been comparatively well studied in atherosclerosis and have direct or indirect links to macrophage cholesterol handling. Apoptosis may support plaque homeostasis when apoptotic cells are efficiently cleared by efferocytosis, thereby limiting inflammation and promoting LXR-dependent cholesterol efflux. In contrast, defective efferocytosis can lead to secondary necrosis and inflammatory amplification. Pyroptosis, necroptosis, and ferroptosis may impair macrophage cholesterol homeostasis through overlapping mechanisms involving inflammatory signaling, oxidative stress, membrane disruption, reduced ABCA1/ABCG1-dependent efflux, and HDL dysfunction. Autophagy and lipophagy exert context-dependent effects: appropriate autophagic flux facilitates intracellular cholesterol mobilization, whereas excessive or dysregulated autophagy may increase ferroptotic susceptibility through ferritinophagy, iron release, and GPX4 loss. Based on these observations, we propose a macrophage death-mode bifurcation framework centered on caspase-8 activity, RIPK1 ubiquitination, mitochondrial stress, oxidative stress thresholds, and autophagic flux. These interconnected nodes may influence whether macrophages undergo apoptosis with efficient clearance or inflammatory and lytic death associated with impaired net cholesterol flux. However, the strength of evidence differs among death modalities, and many proposed links remain indirect or primarily supported by preclinical studies. This framework provides a basis for future studies integrating cell-death regulation, cholesterol transport, and plaque stability.

## Introduction

Atherosclerosis (AS) is a chronic progressive disease of the arterial wall characterized by lipid deposition, chronic inflammatory responses, and the formation of fibrolipid plaques. It represents the primary pathological basis for cardiovascular events such as myocardial infarction and ischemic stroke. During the development and progression of AS, multiple factors including dyslipidemia, vascular endothelial dysfunction, and immune-inflammatory responses interact to cause low-density lipoprotein (LDL) retention and oxidative modification within the arterial intima. This further induces monocyte recruitment, macrophage activation, and lipid deposition, ultimately forming complex atherosclerotic plaque structures (1).

Lipid-lowering therapy, represented by statins, has significantly reduced low-density lipoprotein cholesterol (LDL-C) levels and effectively decreased the incidence and mortality of atherosclerotic cardiovascular disease (ASCVD) (2). However, substantial residual cardiovascular risk persists even among patients receiving intensive lipid-lowering therapy and achieving recommended LDL-C targets.suggesting that simply lowering LDL-C levels is insufficient to completely halt the progression of atherosclerosis (3).

Statins reduce cardiovascular risk across a broad range of patient populations, but they do not eliminate atherosclerotic events. The magnitude of benefit varies according to baseline cardiovascular risk, treatment intensity and duration, medication adherence, and the absolute reduction in LDL-C achieved. Interindividual variation in statin pharmacokinetics, drug tolerance, and LDL-receptor-related pathways may further contribute to heterogeneous lipid-lowering responses. Importantly, cardiovascular events occurring during statin therapy do not necessarily indicate an absence of treatment benefit. Rather, substantial residual risk may persist through pathways not fully corrected by LDL-C lowering, including lipoprotein(a), triglyceride-rich remnant particles, diabetes, smoking, thrombosis, chronic inflammation, and impaired HDL-mediated cholesterol transport (2–4).

Among these residual pathways, impaired macrophage cholesterol handling is particularly relevant to the present review because it links lipid accumulation, inflammation, and regulated cell death within the plaque microenvironment. Reverse cholesterol transport (RCT) is a key pathway by which the body transports excess cholesterol from peripheral tissues to the liver for ultimate excretion, playing a protective role in maintaining cholesterol homeostasis and inhibiting atherosclerosis progression (5). Within AS plaques, macrophages gradually transform into foam cells through uptake of modified lipoproteins, and macrophage-mediated cholesterol efflux represents the initial and rate-limiting step of RCT. Inefficient cholesterol efflux promotes persistent lipid accumulation and foam-cell formation (6). At the lesion level, sustained cholesterol retention, inflammation, and macrophage loss contribute to necrotic-core expansion and plaque instability (7).

Within atherosclerotic plaques, circulating monocytes differentiate into macrophages and internalize modified lipoproteins, progressively forming foam cells. When cholesterol efflux is insufficient, membrane and intracellular sterol accumulation can enhance Toll-like receptor signaling and inflammatory activation (8). Cholesterol crystals provide a more direct stimulus for NLRP3 inflammasome activation and IL-1β production (9). Macrophages also remove apoptotic cells through efferocytosis, a process that contributes to inflammation resolution and tissue repair (10). Under chronic inflammatory conditions, however, clearance capacity may become insufficient, allowing cellular debris and cholesterol to accumulate. These interactions among lipid loading, inflammatory signaling, and dead-cell clearance form a metabolic–inflammatory network that may influence both local cholesterol flux and plaque stability.

However, a critical gap exists in current research: although the molecular mechanisms of these four death modes have been extensively described individually, how they form a “bifurcation decision system” through shared signaling nodes and collectively determine RCT efficiency remains systematically unexplored. Current literature predominantly discusses the relationship between each single death mode and cholesterol metabolism, overlooking a core fact—within the AS plaque microenvironment, cells do not “selectively” undergo a specific type of death, but rather undergo dynamic bifurcation at key molecular switches such as caspase family members, ubiquitination modification systems, and mitochondrial signals. The direction of this proposed bifurcation may influence whether stressed macrophages undergo apoptosis followed by efficient efferocytosis or shift toward inflammatory and lytic death associated with impaired cholesterol handling. We therefore propose that crosstalk among macrophage death modalities represents one regulatory layer that may shape net cholesterol flux and plaque outcome. Although a growing number of regulated cell-death modalities have been described, this review focuses on apoptosis, pyroptosis, necroptosis, and ferroptosis for three main reasons. First, these modalities have been relatively well characterized in plaque macrophages and are supported by mechanistic or *in vivo* evidence in atherosclerosis. Second, each has been linked, either directly or indirectly, to macrophage cholesterol handling, efferocytosis, inflammatory signaling, or HDL function. Third, they converge on shared regulatory nodes—including caspase-8, RIPK1 ubiquitination, mitochondrial stress, inflammasome activation, and oxidative stress—which makes them particularly suitable for the proposed death-mode bifurcation framework. Other forms of regulated cell death, including cuproptosis, parthanatos, and oxeiptosis, may also contribute to atherosclerosis; however, direct evidence connecting these processes to macrophage cholesterol efflux or whole-body reverse cholesterol transport remains comparatively limited. NETosis is also relevant to plaque inflammation but primarily involves neutrophils and therefore lies outside the macrophage-centered scope of this review.

Accordingly, this review integrates the crosstalk among these death modalities and evaluates how it may influence net cholesterol flux through changes in cholesterol uptake, intracellular mobilization, transporter-dependent efflux, HDL function, and efferocytic clearance.

## 2 Macrophage cholesterol flux and reverse cholesterol transport

### 2.1 Cholesterol influx and lipoprotein uptake by macrophages

Macrophage cholesterol homeostasis reflects the net balance among modified-lipoprotein uptake, intracellular esterification and mobilization, transporter-mediated efflux, and lipid transfer during efferocytosis. Modified lipoproteins are predominantly internalized through scavenger receptors, including CD36, SR-A1, and LOX-1. Inflammatory and oxidative signaling associated with lytic cell-death programs may alter the expression or activity of these receptors; however, direct evidence that an individual death program specifically increases macrophage cholesterol influx remains limited. SR-BI warrants separate consideration because it mediates bidirectional cholesterol movement. Depending on the cholesterol gradient, HDL composition, and cellular context, SR-BI may facilitate free-cholesterol efflux while also mediating selective uptake of HDL-derived cholesteryl esters. Therefore, changes in SR-BI expression should be interpreted in terms of net intracellular cholesterol balance rather than being classified exclusively as changes in either influx or efflux.

### 2.2 Cholesterol efflux and HDL maturation

Cholesterol efflux represents the initial step of reverse cholesterol transport (RCT), transferring excess cholesterol from peripheral tissues to circulating high-density lipoprotein (HDL), initiating the clearance process toward the liver. In atherosclerotic lesions, this process primarily occurs in macrophage foam cells formed following uptake of oxidized low-density lipoprotein, suggesting that macrophage cholesterol efflux is the key step of RCT.

The ATP-binding cassette transporters A1 (ABCA1) and G1 (ABCG1) on cell membranes are the primary carriers mediating cholesterol efflux (11). ABCA1 promotes the transfer of free cholesterol and phospholipids to lipid-poor apolipoprotein AI (apoA-I), generating nascent HDL; ABCG1 further transfers cholesterol to mature HDL particles, exhibiting functional complementarity in the cholesterol efflux process (8). Experimental studies demonstrate that ABCA1 and ABCG1 have complementary roles in macrophage cholesterol export. Combined deficiency of these transporters markedly increases foam-cell lipid accumulation and accelerates atherosclerosis, indicating that loss of both pathways produces a more severe phenotype than impairment of either pathway alone (12).

After cholesterol is transferred to the HDL surface through ABCA1/ABCG1-mediated transport, HDL must further mature to continuously drive reverse cholesterol transport. The core process of HDL maturation is cholesterol esterification, primarily mediated by lecithin-cholesterol acyltransferase (LCAT) in plasma. LCAT catalyzes the esterification reaction between free cholesterol and phosphatidylcholine on the HDL surface, generating more hydrophobic cholesterol esters that are transferred to the hydrophobic core of HDL, converting nascent discoidal HDL into more stable spherical mature HDL (13). Mature HDL can more effectively maintain the cholesterol concentration gradient between cells and plasma, continuously promoting cholesterol efflux. apoA-I not only serves as the primary structural protein of HDL providing structural support, but also enhances LCAT enzymatic activity, making HDL maturation more efficient. LCAT or apoA-I dysfunction leads to impaired HDL maturation and affects overall RCT efficiency, emphasizing the importance of the HDL maturation process in maintaining cholesterol homeostasis (14).

### 2.3 HDL transport to the liver and cholesterol excretion

Mature HDL particles transport cholesterol transferred from peripheral cells to the liver, representing a critical link in RCT. HDL cholesterol transport to the liver primarily involves two complementary pathways: the direct pathway and the indirect pathway. The direct pathway is mediated by SR-BI on hepatocyte surfaces, facilitating HDL binding to hepatocyte membranes and enabling selective uptake of HDL core cholesterol esters without altering the overall HDL particle structure. This mechanism efficiently transfers cholesterol loaded on HDL to hepatocytes and represents one of the major pathways in the terminal stage of RCT (15).

The indirect pathway primarily relies on cholesteryl ester transfer protein (CETP)-mediated lipoprotein exchange. In humans and certain animal models, CETP can transfer cholesterol esters from HDL to very low-density lipoprotein (VLDL) or low-density lipoprotein (LDL), after which these lipoproteins are taken up by hepatocytes through LDL receptors (LDLR) on the hepatocyte surface, mediating transport to the liver (16). Although CETP plays an important regulatory role in lipoprotein metabolism, its effects on overall RCT efficiency and atherosclerosis protection show complexity in clinical studies and remain controversial.

After completion of transport to the liver, cholesterol must be effectively excreted from the body to achieve true clearance. The liver excretes the taken-up cholesterol through two major pathways: conversion to bile acids or direct excretion into the intestine via bile. Cholesterol 7*α*-hydroxylase (CYP7A1) is the rate-limiting enzyme in the classical bile acid synthesis pathway. By catalyzing the 7*α*-hydroxylation reaction of cholesterol, it converts cholesterol into bile acid precursors (17). The expression and enzymatic activity of CYP7A1 are key factors affecting the efficiency of cholesterol conversion to bile acids. Studies indicate that enhanced CYP7A1 activity significantly increases bile acid synthesis and secretion, promoting cholesterol excretion and maintaining systemic cholesterol homeostasis. Another excretion pathway is the direct excretion of cholesterol as neutral sterols into the intestine via bile. This process primarily relies on the ATP-binding cassette transporters G5/G8 (ABCG5/ABCG8) heterodimer to transport intracellular cholesterol in hepatocytes to the bile canaliculus membrane and enter the bile (18). Expression of ABCG5/ABCG8 significantly increases biliary cholesterol secretion and represents one of the important mechanisms for hepatic cholesterol clearance.

These two excretion mechanisms collectively ensure that cholesterol transported to the liver via HDL can ultimately leave the body. Among them, the CYP7A1-mediated bile acid generation pathway represents the major degradation pathway for cholesterol metabolism, while the ABCG5/ABCG8-mediated direct biliary excretion pathway assists in enhancing cholesterol clearance efficiency (5). In SR-BI-deficient mice, hepatic PON1 expression improved HDL functionality and promoted hepatic cholesterol uptake through an alternative APOE–LDLR-related pathway (19). Therefore, macrophage cholesterol efflux capacity, HDL structural and functional status, and hepatic clearance pathways collectively construct the overall effectiveness of RCT, and functional imbalance at any link may weaken reverse cholesterol transport and promote atherosclerosis progression.

### 2.4 Integration of cholesterol influx, mobilization, and efflux

The core of RCT lies in macrophage-mediated cholesterol efflux to HDL through ATP-binding cassette transporters ABCA1 and ABCG1, which is a key mechanism for maintaining cholesterol homeostasis. On this basis, RCT efficiency is also subject to enhanced regulation at multiple levels, and these regulatory mechanisms collectively determine the functional state of macrophages within the plaque microenvironment.

Macrophage cholesterol efflux is closely linked to immune homeostasis. ABCA1/ABCG1-mediated cholesterol removal can remodel membrane lipid rafts and reduce signaling through receptors such as TLR4, whereas transporter deficiency promotes membrane sterol accumulation and inflammatory activation (8). Cholesterol accumulation may also amplify inflammatory responses beyond the macrophage itself; in experimental atherosclerosis, macrophage cholesterol loading promoted IL-1β release and neutrophil extracellular trap formation (20). These observations support reciprocal interactions between cholesterol retention and inflammation, but they do not establish that reduced ABCA1/ABCG1 expression uniformly causes oxidative cell death in all plaque macrophage subsets.

The pathological consequences of cell death also depend on the capacity for clearance of dead cells within plaques—that is, efferocytosis. Normal efferocytosis can promptly clear apoptotic cells (particularly apoptotic foam cells), inhibit inflammatory cascades, and promote tissue repair, maintaining plaque stability. Conversely, when sustained cell death is accompanied by impaired efferocytosis, it leads to secondary necrosis, release of pro-inflammatory factors, expansion of the necrotic core, and thinning of the fibrous cap, significantly increasing the risk of plaque rupture and acute cardiovascular events (21). Preclinical and review studies indicate that efferocytosis defects are key drivers of plaque progression and instability, and they overlap with RCT pathway impairments (such as decreased ABCA1/ABCG1 function), forming a positive feedback loop that promotes inflammation and monocyte/macrophage recruitment.

In summary, cholesterol accumulation and cholesterol efflux capacity jointly determine the survival fate of macrophages within plaques: on one hand, cholesterol overload promotes cell death through mitochondrial destabilization and oxidative stress; on the other hand, lCholesterol overload promotes cell death through mitochondrial destabilization and oxidative stress, whereas ABCA1/ABCG1-mediated lipid efflux exerts cytoprotective and anti-inflammatory effects; Cholesterol overload promotes cell death through mitochondrial destabilization and oxidative stress, whereas ABCA1/ABCG1-mediated lipid efflux exerts cytoprotective and anti-inflammatory effects.

Meanwhile, efferocytosis capacity ultimately determines the pathological consequences of these death events. However, macrophage death in the context of lipid overload and complex inflammatory microenvironments is not a singular pathological process. Different programmed cell death pathways not only determine the degree of pro-inflammatory content release but also directly impact the recognition and clearance efficiency of efferocytosis receptors. Therefore, systematically elucidating the roles of these macrophage death modes in reverse cholesterol transport and identifying their core regulatory nodes will provide new potential targets for intervening in reverse cholesterol transport and modulating atherosclerosis.

## 3 Macrophage programmed cell death modes

Macrophage death is shaped by the lipid-rich, oxidizing, and inflammatory microenvironment of atherosclerotic plaques. Its pathological consequences depend not only on the molecular execution pathway but also on membrane integrity, the efficiency of dead-cell clearance, the availability of viable cholesterol-exporting macrophages, and the functional state of extracellular cholesterol acceptors. This section therefore summarizes the pathway-specific features of apoptosis, pyroptosis, necroptosis, and ferroptosis, together with the strength of evidence linking each modality to macrophage cholesterol handling. Shared regulatory nodes, pathway switching, and the integrated consequences of death-mode crosstalk are discussed in Section 5.

### 3.1 Macrophage apoptosis

#### 3.1.1 Stage-dependent effects of apoptosis and efferocytosis

The effect of macrophage apoptosis on atherosclerosis is strongly dependent on lesion stage and local efferocytic capacity. In early lesions, controlled apoptosis may restrict the accumulation of damaged or lipid-overloaded macrophages when apoptotic cells are rapidly recognized and removed by neighboring phagocytes. Experimental suppression of macrophage apoptosis can paradoxically accelerate early lesion development when clearance remains efficient, indicating that apoptosis is not intrinsically proatherogenic (22). More broadly, the balance between the generation and clearance of apoptotic cells is a major determinant of lesion progression and plaque stability (7).

Efferocytosis links apoptotic-cell clearance to cholesterol metabolism because the recipient phagocyte acquires the lipid and cholesterol content of the ingested cell. Apoptotic-cell-derived cholesteryl esters are delivered to lysosomes, where hydrolysis releases free cholesterol and generates oxysterols capable of activating liver X receptor (LXR) signaling. This adaptive response induces ABCA1 and ABCG1 and promotes cholesterol transfer to apoA-I and HDL, thereby coupling dead-cell clearance to intracellular sterol disposal rather than passive lipid accumulation (23). Efferocytosis can additionally activate lysosome-dependent LXR and PPAR*δ* signaling in human macrophages, supporting coordinated metabolic and anti-inflammatory adaptation (24).

These findings support a local relationship among efferocytosis, lysosomal cholesterol processing, LXR activation, and macrophage cholesterol efflux. However, they should not be interpreted as direct proof that increasing apoptosis uniformly enhances whole-body reverse cholesterol transport (RCT). The beneficial effect requires that apoptotic-cell production remains within local clearance capacity and that lysosomal processing, transporter-dependent efflux, and extracellular acceptor function remain intact.

#### 3.1.2 Defective efferocytosis and impaired cholesterol disposal

In advanced plaques, apoptosis frequently occurs in the setting of defective efferocytosis. Uncleared apoptotic cells undergo secondary necrosis and release intracellular lipids, proteases, oxidized molecules, and damage-associated molecular patterns (DAMPs). These products amplify inflammation, recruit additional immune cells, enlarge the necrotic core, and destabilize the plaque (7, 21). Defective clearance also eliminates the lysosome-dependent oxysterol response that normally helps recipient macrophages accommodate apoptotic-cell-derived cholesterol (23).

Persistent inflammation can further impair cholesterol handling in surviving macrophages. In oxLDL-loaded macrophages, NF-*κ*B-associated signaling has been linked to reduced ABCA1 expression and enhanced inflammasome-dependent death (25). Because ABCA1 and ABCG1 mediate cholesterol transfer to apoA-I and HDL, reduced transporter abundance or activity favors sterol retention and foam-cell persistence (8). Nevertheless, current evidence does not demonstrate that apoptosis itself directly represses these transporters. A more defensible interpretation is that defective efferocytosis, secondary necrosis, and the resulting inflammatory environment indirectly compromise cholesterol export.

The acidic microenvironment of advanced plaques provides a more specific post-translational mechanism. Extracellular acidification activates acid-sensing ion channel 1-dependent Ca^2+^ influx and calpain-1, accelerating ABCA1 proteolysis and reducing ABCA1-mediated cholesterol efflux (26). ABCA1 contains a PEST sequence that confers sensitivity to calpain-mediated degradation, whereas interaction with apoA-I stabilizes ABCA1 at the plasma membrane (27). AMPK activation can also enhance ABCA1 stability by retarding calpain-mediated degradation (28). Proteolysis is therefore an established form of post-translational regulation in this setting. By contrast, direct effects of extracellular acidification on ABCA1 phosphorylation, ubiquitination, or glycosylation have not been demonstrated and should remain identified as unresolved possibilities.

Advanced inflammation also compromises the extracellular acceptor component of cholesterol transport. Serum amyloid A enrichment and other acute-phase changes remodel the HDL proteome (29), and inflammatory HDL remodeling is associated with reduced macrophage cholesterol efflux capacity (30). HDL particles isolated under different pathological conditions display substantial variation in cholesterol-accepting function that is not captured by HDL-C concentration alone (31). Myeloperoxidase-dependent apoA-I oxidation has likewise been directly linked to impaired ABCA1-dependent efflux in patients with atherosclerosis (32).

Thus, the pathological effect of macrophage apoptosis is determined primarily by whether the apoptotic cells are efficiently cleared. Successful efferocytosis supports controlled lipid transfer and adaptive cholesterol export, whereas failed clearance promotes secondary necrosis, inflammation, HDL dysfunction, cholesterol retention, and necrotic-core expansion.

### 3.2 Pyroptosis

#### 3.2.1 Core execution and activation by cholesterol overload

Pyroptosis is a lytic and pro-inflammatory form of regulated cell death characterized by gasdermin-dependent membrane pore formation. In the canonical pathway, inflammasome-associated caspase-1 processes pro-IL-1β and pro-IL-18 and cleaves gasdermin D (GSDMD). The non-canonical pathway is initiated by caspase-4/5 in humans or caspase-11 in mice and likewise converges on GSDMD cleavage (33–36). GSDMD-mediated mitochondrial permeabilization and mitochondrial ROS generation may amplify pyroptotic signaling, although the broader significance of mitochondrial crosstalk is discussed in Section 5 (37).

Cholesterol crystals provide one of the best-established links between cholesterol accumulation and inflammasome activation in atherosclerosis. Following phagocytosis, crystals damage lysosomal membranes, release cathepsins, and promote NLRP3-dependent IL-1β maturation. Genetic and pharmacological studies support a causal contribution of this pathway to crystal-induced inflammation and experimental atherogenesis (9, 38, 39).

OxLDL can reinforce this response through scavenger-receptor-mediated uptake and intracellular crystal formation. CD36 coordinates the internalization and conversion of soluble ligands into particulate material, thereby connecting modified-lipoprotein uptake to lysosomal stress and NLRP3 activation (40). TLR/NF-*κ*B signaling provides inflammasome priming, whereas potassium efflux, mitochondrial injury, ROS, and defective autophagic clearance may lower the activation threshold. HDL can oppose this axis by suppressing cholesterol-crystal-induced inflammasome activation and IL-1β release (41).

#### 3.2.2 Pyroptosis and reverse cholesterol transport

Pyroptotic membrane rupture has direct consequences for macrophage cholesterol handling. Once lysis is completed, the dying macrophage can no longer sustain regulated cholesterol export, and intracellular lipids are redistributed into the extracellular plaque compartment. Passive lipid release from a ruptured cell is not equivalent to productive ABCA1/ABCG1-dependent efflux to apoA-I or HDL and does not constitute RCT.

Among the death modalities considered in this review, pyroptosis has the strongest direct evidence for an effect on systemic macrophage RCT. Opoku et al. demonstrated that GSDMD mediated inflammation-induced reductions in macrophage cholesterol efflux and impaired the movement of macrophage-derived cholesterol to plasma, liver, and feces. GSDMD deficiency partially preserved these processes and reduced atherosclerotic lesion burden (42). This study is particularly informative because it assessed cholesterol movement across multiple systemic compartments rather than relying solely on transporter abundance.

At the cellular level, activation of the NF-*κ*B/ABCA1 axis has been associated with oxLDL-induced NLRP3 activation and pyroptosis in THP-1-derived macrophages, whereas pathway inhibition restored ABCA1 expression and reduced inflammatory cell death (25). These observations support reciprocal reinforcement between cholesterol retention and inflammasome signaling. However, the evidence is strongest for ABCA1, and pyroptosis should not be described as directly repressing all major cholesterol transporters without transporter-specific evidence.

IL-1β and IL-18 released during inflammasome activation contribute to leukocyte recruitment, endothelial activation, and inflammatory amplification. The CANTOS trial demonstrated that IL-1β inhibition reduced recurrent cardiovascular events independently of LDL-C lowering, confirming the clinical relevance of inflammatory residual risk (43, 44). CANTOS did not, however, directly assess macrophage cholesterol efflux or whole-body RCT and should not be cited as direct evidence that IL-1β blockade restores these processes.

Pyroptosis-associated inflammation may also impair the extracellular acceptor side of cholesterol transport. Acute-phase remodeling, oxidation, and compositional changes can reduce HDL-mediated cholesterol efflux even when HDL-C concentration is unchanged (30, 32, 45). GSDME-mediated lytic death provides an additional inflammatory pathway in atherosclerosis, as GSDME deficiency reduced lesion area and inflammatory signaling in ApoE-deficient mice (46). Nevertheless, the direct effects of GSDME on macrophage cholesterol efflux and systemic RCT remain undefined.

Overall, pyroptosis may impair cholesterol transport through loss of viable efflux-competent macrophages, membrane disruption, inflammatory suppression of cellular efflux pathways, and deterioration of HDL acceptor quality. Of these relationships, the GSDMD–RCT connection is supported most directly.

### 3.3 Necroptosis

#### 3.3.1 RIPK1–RIPK3–MLKL signaling and plaque evidence

Necroptosis is a regulated lytic death pathway centered on receptor-interacting protein kinase 1 (RIPK1), RIPK3, and mixed lineage kinase domain-like protein (MLKL). When caspase-8-mediated apoptotic restraint is insufficient, RIPK1 and RIPK3 can form a necrosome, leading to RIPK3-dependent MLKL phosphorylation, oligomerization, and membrane injury (47). Because caspase-8 activity and RIPK1 ubiquitination also regulate apoptosis and inflammasome-associated signaling, their shared roles are discussed in detail in Section 5.

Lipid stress may sensitize plaque macrophages to necroptosis. OxLDL and cholesterol accumulation increase oxidative stress, endoplasmic-reticulum stress, TLR signaling, and TNF-family cytokine production. In a macrophage model, PCB29-pQ increased cellular cholesterol and cholesteryl esters while activating RIPK1/RIPK3/MLKL-dependent death, providing mechanistic support for an association between disturbed cholesterol balance and necroptosis (48). Because this model used an environmental toxicant, it should not be interpreted as evidence that the same pathway predominates in human plaques.

More direct evidence comes from atherosclerosis models. RIPK3-dependent macrophage necrosis promotes lesion development, whereas hematopoietic RIPK3 deficiency reduces advanced atherosclerosis and necrotic-core formation (49). Pharmacological or genetic targeting of macrophage necroptosis likewise reduces lesion inflammation and progression (50). RIPK1 expression is associated with inflammatory activity in early human atherosclerosis, and RIPK1 silencing reduces NF-*κ*B activation and atherogenesis in mice (51).

#### 3.3.2 Consequences for cholesterol handling and plaque stability

The effect of necroptosis on cholesterol transport is biologically plausible but less directly established than that of GSDMD-dependent pyroptosis. MLKL-mediated membrane rupture releases intracellular cholesterol, oxidized lipids, ATP, HMGB1, and other DAMPs into the plaque. These materials may be re-internalized by neighboring macrophages and increase local lipid burden. DAMP-induced inflammatory signaling may additionally reduce ABCA1 expression, promote inflammatory uptake pathways, and impair efferocytic disposal.

However, direct experiments demonstrating that MLKL activation selectively downregulates ABCA1 or ABCG1 in plaque macrophages remain limited. The most rigorous interpretation is that necroptosis indirectly favors cholesterol retention through three overlapping consequences: loss of viable efflux-competent macrophages, extracellular redistribution of cellular lipids, and inflammation-mediated reprogramming of neighboring cells.

Necroptosis contributes more clearly to plaque instability. RIPK3- and MLKL-associated macrophage loss promotes necrotic-core expansion, inflammatory recruitment, and matrix degradation (49, 50). Oxidative stress, neutrophil extracellular traps, and macrophage necroptosis may further amplify injury in advanced plaques (52). Nevertheless, the release of DAMPs, phosphatidylserine exposure, and procoagulant material is not unique to necroptosis. Pathway attribution therefore requires genetic, pharmacological, and pathway-specific molecular validation rather than morphology alone.

The most robust conclusion is that RIPK1/RIPK3/MLKL signaling promotes inflammatory cell loss, necrotic-core formation, and plaque progression. Its direct quantitative effects on macrophage cholesterol efflux and whole-body RCT remain unresolved.

### 3.4 Ferroptosis

#### 3.4.1 Core machinery and interaction with foam-cell metabolism

Ferroptosis is an iron-dependent form of regulated cell death driven by the accumulation of phospholipid hydroperoxides rather than caspase activation or gasdermin pore formation (53). The System Xc−–glutathione–GPX4 axis constitutes a principal antioxidant defense, whereas cellular iron availability and the incorporation of polyunsaturated fatty acids into membrane phospholipids determine the abundance of oxidizable substrates (54). Reduced GPX4, increased ACSL4, lipid ROS, or iron accumulation alone is not sufficient to establish ferroptotic death. Strong evidence requires pharmacological rescue, genetic manipulation, or convergent biochemical and morphological assessment.

Foam-cell formation changes the lipid and redox environment in which ferroptosis occurs. Modified-lipoprotein uptake increases cholesteryl-ester storage, free cholesterol in cellular membranes, oxidized fatty-acid species, lysosomal stress, and mitochondrial dysfunction. Although cholesterol is not itself the principal substrate of ferroptotic lipid peroxidation, cholesterol overload may increase susceptibility by altering membrane organization, organelle function, oxidative pressure, and the abundance of peroxidizable phospholipids.

Impaired ABCA1/ABCG1 function may reinforce this environment by promoting sterol retention, membrane-raft remodeling, TLR signaling, and ROS generation. Conversely, reducing intracellular lipid burden may indirectly improve resistance to ferroptosis. Direct evidence that ABCA1 or ABCG1 functions as a core ferroptosis regulator remains limited; the available data support an indirect association mediated by lipid burden, membrane composition, and redox state rather than a fully established ABCA1/ABCG1–ferroptosis axis.

High HMOX1 expression under hypoxic conditions increases iron-dependent oxidative stress and promotes ferroptosis in macrophage-derived foam cells, accompanied by greater plaque burden and instability (55). This finding illustrates the context dependence of iron-handling pathways: moderate haem oxygenase activity may be cytoprotective, whereas excessive induction may increase the labile iron pool. Antiferroptotic therapy also attenuates atherosclerotic development and oxidative injury in experimental models, supporting a causal contribution of ferroptosis to lesion progression (56).

#### 3.4.2 Plaque instability and the evidence gap in RCT

Ferroptotic macrophages contribute oxidized phospholipids, iron, and cellular debris to the plaque, particularly when dead-cell clearance is inadequate. This redox-active environment may promote further lipid peroxidation, inflammatory recruitment, matrix-degrading enzyme expression, and necrotic-core expansion (55, 57).

Ferroptosis in vascular smooth muscle cells may also weaken the fibrous cap by reducing the number of viable matrix-producing cells. YAP1-dependent regulation of GLS1 suppresses vascular smooth muscle cell ferroptosis and enhances plaque stability, providing evidence that ferroptotic signaling can influence plaque structure rather than merely serving as a molecular marker (58).

The relationship between ferroptosis and RCT is less direct. Ferroptotic cell loss reduces the population capable of regulated cholesterol export, while lipid-peroxidation products may oxidize neighboring membranes, apoA-I, or HDL. Nevertheless, macrophage-to-feces RCT studies specifically testing ferroptosis are currently lacking. Ferroptosis should therefore be described as being associated with impaired net cholesterol handling and oxidative deterioration of the plaque environment, rather than as an established direct blocker of systemic RCT.

## 4 Roles of autophagy and lipophagy in cholesterol metabolism

Autophagy is a lysosome-dependent quality-control and recycling system, whereas lipophagy refers to the selective delivery of lipid droplets to lysosomes. In atherosclerotic plaque cells, these processes influence intracellular cholesterol mobilization, organelle quality control, inflammatory activation, and susceptibility to regulated cell death. This section focuses on autophagic flux and cholesterol mobilization; the interfaces through which autophagy modifies apoptosis, pyroptosis, necroptosis, and ferroptosis are integrated in Section 5.

### 4.1 Autophagic flux and lipid-droplet processing

Autophagy involves ULK1-dependent initiation, class III phosphatidylinositol 3-kinase-dependent membrane nucleation, ATG12–ATG5–ATG16L1-mediated membrane expansion, LC3/GABARAP lipidation, autophagosome closure, and fusion with lysosomes (59–61). For atherosclerosis research, however, assessment of complete autophagic flux is more informative than measurement of the abundance of a single autophagy marker. Increased LC3-II may reflect either enhanced autophagosome formation or impaired autophagosome clearance, whereas p62/SQSTM1 accumulation may result from defective autophagic degradation or transcriptional upregulation. Therefore, LC3-II and p62 levels should be interpreted together with dynamic autophagic-flux assays rather than being used alone to infer autophagy activation or inhibition (62).

Autophagic competence changes during lesion development. Adaptive autophagy can remove damaged organelles and mobilize intracellular lipid stores, whereas chronic lipid loading, oxidative injury, and lysosomal dysfunction may block late-stage flux. Macrophage autophagy deficiency promotes inflammasome activation and atherosclerotic progression, demonstrating that the biological effect depends on whether the complete degradative pathway remains functional (63).

In foam cells, a substantial fraction of excess cholesterol is stored as cholesteryl esters within cytoplasmic lipid droplets. Ouimet et al. demonstrated that autophagy delivers lipid droplets to lysosomes, where lysosomal acid lipase hydrolyzes cholesteryl esters and supplies free cholesterol predominantly for ABCA1-dependent efflux (64). This provides the principal mechanistic basis for linking lipophagy to macrophage cholesterol export.

Lipophagy complements rather than replaces neutral cholesteryl-ester hydrolases. Following lysosomal hydrolysis, free cholesterol may be transported to the plasma membrane, re-esterified in the endoplasmic reticulum, incorporated into cellular membranes, or converted to oxysterols. Therefore, lipophagy does not automatically guarantee net cholesterol removal; its benefit depends on intracellular trafficking, transporter availability, and functional extracellular acceptors.

Multiple lipid-droplet-associated and autophagy-related proteins, including SQSTM1/p62, NBR1, and OPTN, participate in cargo recognition, trafficking, and lysosomal delivery in macrophage foam cells (6). These findings support a coordinated cargo-recognition network rather than a single universal lipophagy receptor. NCOA4 is best established as a ferritinophagy receptor and should not be presented as a direct lipid-droplet receptor without specific evidence.

Lysosomal competence is essential for productive lipophagy. Cholesterol crystals and oxidized lipids can damage lysosomal membranes, while impaired intracellular cholesterol transport reduces autophagosome–lysosome fusion and blocks cargo degradation (65, 66). Lipid accumulation may therefore both stimulate autophagic responses and impair the lysosomal system required for their completion.

### 4.2 Coupling lipophagy to cholesterol efflux

Lipophagy supplies free cholesterol to downstream transport pathways but does not itself complete RCT. ABCA1 transfers free cholesterol and phospholipids to lipid-poor apoA-I, whereas ABCG1 facilitates cholesterol transfer to mature HDL. Effective cholesterol clearance therefore requires coordination among lysosomal cholesteryl-ester hydrolysis, intracellular trafficking, transporter abundance and localization, and extracellular acceptor function (8, 64).

LXR activation increases ABCA1 and ABCG1 expression, while TFEB-dependent lysosomal biogenesis can enhance degradative capacity and cholesterol mobilization. Activation of the mTORC1/TFEB/ABCA1–SCARB1 axis has been reported to promote lipophagy and cholesterol efflux in experimental atherosclerosis (67). Combined treatment with mangiferin and the LXR agonist T0901317 likewise enhanced lipid-droplet degradation, increased ABCA1/ABCG1 expression, and promoted macrophage cholesterol efflux; inhibition of autophagy attenuated these effects (68). These pharmacological findings support pathway coupling but should not be generalized to imply that every autophagy activator improves RCT.

Autophagic competence may also modify the expression of genes involved in cholesterol influx and export. In HepG2 and HAP1 cells, disruption of ATG5 or ATG7 altered MK-2206-induced LDLR expression and markedly reduced ABCA1 expression; whether this regulatory relationship also operates in plaque macrophages remains unknown (69). Nevertheless, transporter expression should not be equated automatically with net cholesterol flux, because increased LDL receptor expression may enhance cholesterol entry, while increased ABCA1 expression may fail to improve efflux if lipid mobilization or apoA-I availability is limiting.

Listerin provides an example of post-translational regulation connecting lysosomal pathways with transporter stability. Listerin catalyzes K63-linked polyubiquitination of ABCA1 and protects it from ESCRT/lysosomal degradation, thereby promoting macrophage cholesterol efflux (70). This mechanism is relevant to cholesterol export but should not be interpreted as evidence that Listerin is a lipophagy receptor or a direct regulator of pyroptosis.

### 4.3 Autophagy dysfunction and cell-type specificity

Autophagy failure may occur at initiation, cargo recognition, autophagosome maturation, fusion, or lysosomal degradation. Defective intracellular cholesterol trafficking impairs autophagosome–lysosome fusion, while lysosomal injury reduces lysosomal acid lipase activity and limits the conversion of stored cholesteryl esters into free cholesterol available for export (65, 66).

Macrophage-specific ATG5 deficiency increases oxidative stress and apoptosis, impairs recognition of dying cells, and enlarges plaque necrosis in hyperlipidemic mice (71). Autophagy deficiency also promotes inflammasome activation and atherosclerotic progression (63). These genetic studies provide stronger evidence for the protective role of competent autophagy than observations based solely on static LC3 or p62 measurements.

Autophagy is not regulated identically in all plaque-cell lineages. Leukocyte-derived macrophage foam cells retain a greater capacity to induce autophagy in response to lipid loading, whereas vascular smooth muscle cell-derived foam cells display more restricted autophagic responses and distinct cholesterol-efflux profiles (72). Findings from one cell lineage should therefore not be generalized automatically to another.

### 4.4 Context-dependent effects on cell fate

Autophagy should not be classified as uniformly protective or harmful. When flux is complete and cargo selection is appropriate, lipophagy reduces lipid-droplet burden, and organelle quality control limits mitochondrial ROS and inflammasome-activating signals. These effects preserve viable cholesterol-exporting cells and increase resistance to inflammatory and oxidative injury (63, 71).

The effect changes when protective cargo is selectively degraded. Ferritin degradation increases the labile iron pool, while autophagic degradation of GPX4 removes a central ferroptosis-defense enzyme. Copper-dependent autophagic GPX4 degradation can promote ferroptosis (73). Conversely, activation of the SIRT1–autophag*y* axis attenuated excess-iron-induced ferroptotic injury in foam cells, indicating that competent autophagy can also restrain ferroptosis under defined conditions (74).

Incomplete flux represents a third state. Autophagosome accumulation in the setting of lysosomal failure does not produce efficient cargo degradation and may instead increase cellular stress. The net effect of autophagy is therefore determined by flux completeness, cargo selectivity, cell identity, iron burden, antioxidant capacity, and lesion stage. These variables are incorporated into the death-mode crosstalk framework described below.

## 5 Crosstalk among macrophage programmed cell death modes

The preceding sections describe the pathway-specific features of apoptosis, pyroptosis, necroptosis, ferroptosis, and autophagy. In the actual plaque microenvironment, however, macrophages are exposed simultaneously to lipid overload, cholesterol crystals, inflammatory cytokines, oxidative stress, hypoxia, iron accumulation, and defective efferocytosis. These stimuli may render several death programs competent at the same time.

The purpose of this section is therefore not to repeat the complete execution mechanisms described in Sections 3, 4. Instead, it uses a “death-mode bifurcation” framework to examine how shared regulatory nodes influence pathway selection, coexistence, or switching. Particular attention is given to caspase-8, RIPK1 ubiquitination, mitochondrial and lysosomal stress, oxidative status, and autophagic flux. These nodes may determine whether cellular injury remains compatible with apoptosis and efficient efferocytosis or progresses toward inflammatory, oxidative, and lytic death.

The proposed framework does not imply that a single molecular switch deterministically assigns every macrophage to one death mode. Rather, cell fate is likely determined by the combination, intensity, timing, and subcellular localization of multiple signals. The downstream relevance to cholesterol metabolism lies mainly in their integrated effects on macrophage survival, membrane integrity, intracellular cholesterol mobilization, transporter-dependent efflux, HDL acceptor function, and efferocytic lipid clearance.

### 5.1 Caspase-8 and RIPK1 ubiquitination coordinate apoptosis–pyroptosis–necroptosis switching

#### 5.1.1 Caspase-dependent switching between non-lytic and lytic death

Caspase-8 is a major context-dependent interface among apoptosis, necroptosis, and inflammasome-associated death. Sufficient caspase-8 activity can support apoptotic execution while restraining RIPK1/RIPK3/MLKL-dependent necroptosis. Conversely, loss or inhibition of caspase-8 may release this restraint and permit necroptotic or inflammasome-associated signaling (75).

The canonical NLRP3–caspase-1–GSDMD pathway has been summarized in Section 3.2. Its relevance to crosstalk lies in the ability of the inflammasome platform to engage different downstream caspases according to cellular context. Under canonical conditions, ASC recruits caspase-1, resulting in GSDMD-dependent pyroptosis. When caspase-1-dependent signaling is delayed or impaired, ASC may instead recruit caspase-8 and activate apoptotic caspases, redirecting the cellular response away from conventional pyroptosis (76).

Caspase-3 provides another interface between non-lytic and lytic outcomes. In GSDME-expressing cells, caspase-3 cleavage of GSDME generates a pore-forming N-terminal fragment that can convert an initially apoptotic stimulus into membrane-lytic death (77). In atherosclerosis, GSDME is present in macrophage-rich lesions, and GSDME deficiency reduces lesion burden and inflammatory signaling in ApoE-deficient mice (46). Nevertheless, this process is conditional on GSDME abundance and cellular context and should not be described as an obligatory conversion of apoptosis into pyroptosis in all macrophages.

These different outcomes have distinct implications for lipid disposal. Apoptosis followed by efficient efferocytosis preserves plasma-membrane integrity and permits controlled transfer of cellular lipids to recipient phagocytes. Lysosomal processing of this lipid cargo can activate LXR-dependent adaptive responses and support subsequent cholesterol efflux (24). By contrast, GSDMD- or GSDME-mediated membrane rupture releases cholesterol, oxidized lipids, and DAMPs into the plaque, increasing the burden on neighboring macrophages.

Caspase-8 may therefore influence whether cellular lipid is cleared in a controlled manner or redistributed through inflammatory membrane rupture. However, direct evidence that increased caspase-8 activity independently enhances ABCA1/ABCG1 expression, cellular cholesterol efflux, or whole-body RCT remains lacking. Its effects on cholesterol handling are more appropriately considered consequences of cell-fate selection, membrane integrity, inflammation, and efferocytic competence.

#### 5.1.2 RIPK1 ubiquitination as a survival–death bifurcation node

RIPK1 can participate in survival signaling, apoptosis, necroptosis, or inflammatory activation depending on its ubiquitination state, kinase activity, and associated signaling complexes. K63-linked and M1-linked ubiquitin chains stabilize membrane-associated Complex I and facilitate recruitment of TAK1/TAB and LUBAC–NEMO-related signaling components. These interactions promote NF-*κ*B-dependent survival and inflammatory responses. Deubiquitination or remodeling of RIPK1-associated ubiquitin chains permits RIPK1 to leave Complex I and participate in cytosolic death-signaling complexes (78).

The cIAP1/2–LUBAC–OTULIN network contributes to this regulation. cIAP1/2 promotes K63-linked ubiquitination, LUBAC catalyzes M1-linked linear ubiquitination, and OTULIN removes M1-linked chains. Rather than acting as a simple on–off system, this network controls the stability, duration, and composition of RIPK1-associated complexes.

Within plaques, TNF-family signaling, oxidative stress, and lipid overload may modify the context in which RIPK1 complexes assemble. Caspase-8 inhibition can permit RIPK3–MLKL-dependent necroptosis, whereas maintained caspase-8 activity may direct signaling toward apoptosis or restrain RIPK3 activation (75). However, direct evidence that plaque-derived ROS specifically disables cIAP1/2, destabilizes LUBAC, or produces a defined RIPK1 ubiquitin-chain pattern in plaque macrophages remains limited.

RIPK1-associated signaling may affect cholesterol handling indirectly. TNF-α/NF-*κ*B activation can reduce ABCA1 expression and apoA-I-mediated cholesterol efflux in macrophage foam cells (79). RIPK1 expression is also associated with inflammatory activity in human atherosclerosis, whereas RIPK1 silencing reduces NF-*κ*B activation and atherogenesis in mice (51). Nevertheless, no cited study demonstrates that a defined RIPK1 ubiquitin-chain state directly controls ABCA1/ABCG1-dependent cholesterol transport or systemic RCT.

Accordingly, RIPK1 ubiquitination should be presented as an established regulator of survival–death signaling but only a proposed indirect regulator of cholesterol flux. Its metabolic consequences are likely mediated through inflammatory activation, cell survival, membrane rupture, and lipid redistribution.

#### 5.1.3 Pyroptosis–necroptosis interfaces

RIPK3 is not restricted to canonical MLKL-dependent necroptosis. Under selected conditions, it can also influence NLRP3 inflammasome activation. Caspase-8 normally restrains RIPK1/RIPK3-dependent inflammasome signaling; in its absence or inhibition, RIPK3 kinase activity and MLKL may facilitate NLRP3 activation before or independently of terminal necroptotic lysis (80). Conversely, in inhibitor-of-apoptosis-protein-depleted macrophages with intact caspase-8 activity, RIPK3 can support caspase-8-dependent NLRP3–caspase-1 activation independently of MLKL (81).

These findings identify RIPK3 as a conditional interface between necroptotic machinery and inflammasome signaling rather than an obligatory inducer of simultaneous pyroptosis and necroptosis. Necroptotic and pyroptotic cells may additionally reinforce a shared inflammatory environment by releasing HMGB1, extracellular ATP, mitochondrial components, and other DAMPs. These signals can promote NF-*κ*B-dependent priming, ionic disturbance, or NLRP3 activation (39, 81, 82).

MLKL and GSDMD are distinct membrane-disrupting execution proteins. GSDMD- and MLKL-mediated membrane injury displays different pore characteristics and cellular morphologies (83). The presence of both pathways within a lesion therefore does not demonstrate simultaneous “multipore synergy” in the same macrophage. It also does not establish that either protein directly displaces ABCA1 or ABCG1 from the plasma membrane.

The available evidence supports conditional signaling overlap, parallel activation, and inflammatory amplification. It does not yet establish a universal sequential conversion from pyroptosis to necroptosis or from necroptosis to pyroptosis in cholesterol-loaded plaque macrophages. Their effects on cholesterol transport are most plausibly mediated through loss of viable cells, membrane disruption, DAMP release, inflammatory repression of cholesterol-handling pathways, and deterioration of HDL function.

### 5.2 Mitochondrial, lysosomal, and oxidative stress as shared regulatory contexts

#### 5.2.1 Mitochondrial integration and signal amplification

Mitochondrial dysfunction may function as a shared stress platform connecting apoptosis, pyroptosis, necroptosis, and oxidative injury. However, this integration is supported by pathway-specific observations rather than a single unified mitochondrial code.

In intrinsic apoptosis, BAX/BAK-dependent mitochondrial outer membrane permeabilization (MOMP) permits cytochrome c release and apoptosome-dependent caspase activation (84–86). In atherosclerosis-related systems, oxLDL-associated mitochondrial stress can engage apoptotic signaling, while failure to clear apoptotic macrophages favors secondary necrosis and necrotic-core expansion (87).

Mitochondria may also amplify inflammasome-associated death. Oxidized mitochondrial DNA released during mitochondrial dysfunction can bind NLRP3 and promote inflammasome activation (82). Mitochondrial ROS may contribute through redox-sensitive mechanisms, including TXNIP-associated signaling, although the relative contribution of these pathways depends on stimulus and cellular context (88).

GSDMD provides an experimentally supported connection between pyroptotic execution and mitochondrial injury. Its N-terminal fragment can permeabilize mitochondrial membranes, reduce membrane potential, increase mitochondrial ROS, impair oxidative phosphorylation, and promote the release of mitochondrial proteins and DNA (37). These events may amplify inflammatory signaling, but they do not establish an irreversible NLRP3–GSDMD feedback loop in plaque macrophages.

Elevated mitochondrial ROS has also been reported to promote GSDMD association with mitochondrial membranes and increase susceptibility to gasdermin-associated necroptotic death in an inflammatory model (89). Because this finding was not obtained in cholesterol-loaded plaque macrophages, it should be regarded as evidence of mechanistic possibility rather than proof of a dominant atherosclerotic pathway.

Mitochondrial dysfunction can accompany RIPK3–MLKL signaling, but mitochondria are not universally required for necroptotic execution (47). Mitochondrial outputs should therefore be viewed as context-dependent modifiers and amplifiers rather than obligatory downstream steps in all forms of necroptosis.

From a metabolic perspective, mitochondrial dysfunction can reduce ATP availability and impair energy-dependent cholesterol export. Macrophage mitochondrial energy status has been shown to regulate cholesterol efflux, and improvement of mitochondrial function can enhance this process (90). Thus, mitochondrial injury may simultaneously influence death-pathway susceptibility and the capacity of surviving macrophages to export cholesterol.

#### 5.2.2 Lysosomal injury as a point of convergence

Lysosomal dysfunction links inflammatory signaling, intracellular cholesterol mobilization, and iron-dependent oxidative injury. As summarized in Sections 3.2.1, 4.1, cholesterol crystals and oxidized lipids can destabilize lysosomes, whereas impaired intracellular cholesterol trafficking can block autophagosome–lysosome fusion and cargo degradation (9, 38, 66). The relevance of this process to death-mode crosstalk lies not in repeating the established cholesterol crystal–NLRP3 pathway, but in recognizing that a single lysosomal defect may influence several downstream processes simultaneously.

Cathepsin release may facilitate inflammasome activation, impaired lipophagic flux may reduce the generation of free cholesterol available for transporter-dependent efflux, and disturbed ferritin turnover may alter the labile iron pool. Lysosomal failure may therefore increase intracellular cholesterol retention while lowering the threshold for inflammatory or iron-dependent cell injury. However, the strength of evidence differs among these relationships. Lysosomal damage–dependent inflammasome activation and impaired autophagic cholesterol mobilization are relatively well supported, whereas a direct sequence from cholesterol-crystal-induced lysosomal rupture to ferritin-derived iron release and ferroptosis in plaque macrophages has not been demonstrated.

Lysosomal injury should therefore be regarded as a shared regulatory context rather than an established linear pathway linking crystal uptake, pyroptosis, and ferroptosis. Direct validation will require simultaneous assessment of lysosomal integrity, autophagic flux, inflammasome activation, intracellular iron availability, lipid peroxidation, and ferroptosis-specific rescue within the same macrophage system.

### 5.3 Autophagic flux modifies death-pathway susceptibility

Autophagy does not determine a single fixed death outcome. Instead, it modifies the threshold at which macrophages become susceptible to apoptotic, inflammatory, necroptotic, or ferroptotic injury. The direction of this effect depends primarily on whether autophagic flux is completed, which cargo is degraded, and whether lysosomal and antioxidant capacity remain sufficient.

#### 5.3.1 Competent flux increases resistance to cellular stress

When autophagic–lysosomal flux is complete, removal of damaged mitochondria and other dysfunctional organelles limits mitochondrial ROS, cytochrome c release, oxidized mitochondrial DNA accumulation, and inflammasome-activating signals. Lipophagy can simultaneously mobilize lipid-droplet cholesterol for downstream transporter-dependent efflux (64). These processes preserve viable, efflux-competent macrophages rather than directly inhibiting every individual death pathway.

The protective role of competent flux is supported most directly by macrophage-specific genetic studies. Deficiency of macrophage autophagy increases oxidative stress, apoptosis, defective recognition of dying cells, inflammasome activation, plaque necrosis, and atherosclerotic progression (63, 71). Autophagy should therefore be interpreted as a modifier of cellular stress tolerance and clearance competence rather than as a pathway that universally suppresses apoptosis, pyroptosis, or necroptosis.

The Beclin-1–Bcl-2 interaction provides an additional interface between autophagy and apoptosis, but its functional consequence varies according to stimulus intensity and cellular context (91). It should not be interpreted as a universal quantitative switch between the two processes.

#### 5.3.2 Cargo selectivity determines ferroptotic susceptibility

The effect of autophagy may become pro-death when the selected cargo is required for antioxidant or iron-storage capacity. NCOA4-mediated ferritinophagy increases the availability of intracellular iron, whereas autophagic degradation of GPX4 removes a major defense against phospholipid peroxidation (73, 92, 93). These mechanisms provide a basis through which selective autophagy may lower the threshold for ferroptosis.

Conversely, autophagy may reduce ferroptotic injury when it removes damaged organelles and limits oxidative stress. Activation of the SIRT1–autophag*y* axis attenuated excess-iron-induced ferroptotic injury in foam cells, illustrating that autophagy can be protective under conditions in which organelle quality control outweighs ferritin degradation or GPX4 loss (74).

These findings are not necessarily contradictory. The outcome depends on the baseline iron pool, GPX4 reserve, lysosomal competence, antioxidant capacity, and relative contribution of ferritinophagy, mitophagy, and other selective-autophagy pathways. Although ferritinophagy and autophagic GPX4 degradation are mechanistically established in experimental systems, their quantitative contribution to ferroptosis in plaque macrophages remains incompletely defined.

#### 5.3.3 Autophagy modifies necroptotic and inflammasome signaling

Autophagy may influence necroptotic signaling through both protein turnover and signaling-scaffold functions. The p62/SQSTM1 receptor participates in the clearance of ubiquitinated cargo but may also support the assembly or persistence of RIPK1/RIPK3-associated complexes under selected conditions (94). The direction of this interaction depends on flux competence, cargo availability, and the nature of the initiating stimulus.

Incomplete flux may also facilitate inflammasome-associated injury by allowing damaged mitochondria, mitochondrial ROS, oxidized mitochondrial DNA, and lysosomal cargo to accumulate. This provides a mechanistic explanation for the association between macrophage autophagy deficiency, inflammasome activation, and atherosclerotic progression (63, 95).

Nevertheless, direct evidence that p62 accumulation or autophagy deficiency selectively represses ABCA1 or ABCG1 through a single necroptotic or inflammasome-dependent pathway remains lacking. Effects on cholesterol transport are more plausibly mediated through altered lipid mobilization, mitochondrial function, inflammatory signaling, cell survival, and lysosomal competence.

#### 5.3.4 A context-dependent optimal window

The proposed “optimal window” of autophagy should be regarded as a working model rather than a universal quantitative threshold. Its net effect depends on flux completion, cargo selectivity, lysosomal function, intracellular lipid and iron burden, antioxidant reserve, cell lineage, and lesion stage.

Competent lipophagy and organelle quality control generally favor cholesterol mobilization and stress resistance. In contrast, excessive ferritinophagy, autophagic GPX4 degradation, or incomplete lysosomal flux may increase ferroptotic or inflammatory susceptibility. Thus, the same intervention that enhances autophagy may produce different outcomes in macrophages with different iron loads, lysosomal capacities, or antioxidant states.

Future studies should distinguish autophagy initiation from completed flux and separately quantify lipophagy, mitophagy, ferritinophagy, and GPX4 degradation. Autophagy is therefore best positioned within the proposed bifurcation framework as a context-dependent modifier of death-pathway probability rather than as an exclusively protective or harmful mechanism.

### 5.4 Integrated consequences of death-mode crosstalk for net macrophage cholesterol flux

The consequences of macrophage death-mode crosstalk are best understood at the level of net cellular cholesterol flux rather than changes in a single transporter. Macrophage cholesterol burden reflects the balance among modified-lipoprotein uptake, intracellular esterification and mobilization, transporter-dependent efflux, extracellular acceptor function, and lipid transfer during efferocytosis.

Crosstalk among apoptosis, pyroptosis, necroptosis, ferroptosis, and autophagy may disturb several of these processes simultaneously. The resulting phenotype depends on whether cells remain viable, whether their membranes and organelles remain functional, whether cholesterol can be mobilized from intracellular stores, and whether cellular lipid is cleared through efferocytosis or released into the extracellular plaque compartment.

#### 5.4.1 Cholesterol entry and redistribution

Inflammatory and oxidative signals produced during lytic or oxidative death may increase intracellular cholesterol accumulation through enhanced entry or redistribution of lipid. NF-*κ*B activation, oxidized lipids, and DAMP signaling may increase scavenger-receptor activity in neighboring macrophages. In parallel, extracellular lipid released from ruptured or secondarily necrotic cells can be re-internalized and contribute to secondary foam-cell formation.

However, evidence that a specific death program directly increases modified-lipoprotein uptake remains limited. Increased influx should therefore be considered a context-dependent and partly indirect consequence of inflammatory signaling and lipid redistribution rather than a uniform property of pyroptosis, necroptosis, or ferroptosis.

SR-BI also requires careful interpretation because it mediates bidirectional cholesterol movement. Depending on HDL composition, the cholesterol gradient, and cellular context, SR-BI may support free-cholesterol efflux or selective uptake of HDL-derived cholesteryl esters. Changes in SR-BI during cell stress should therefore be interpreted according to net intracellular cholesterol balance rather than classified exclusively as increased influx or reduced efflux.

#### 5.4.2 Viability and donor-side efflux capacity

Regulated cholesterol export requires viable macrophages with intact membranes, sufficient ATP, functional intracellular trafficking, and accessible cholesterol pools. Lytic death reduces the number of cells capable of regulated efflux. Passive lipid release from pyroptotic or necroptotic cells does not constitute productive RCT.

Among the pathways reviewed here, GSDMD-dependent pyroptosis has the strongest direct evidence for impaired systemic RCT (42). NF-*κ*B-associated inflammation has also been linked to reduced ABCA1 expression in oxLDL-loaded macrophages (25). In contrast, evidence that MLKL activation or ferroptotic execution directly suppresses ABCA1/ABCG1 remains limited.

Mitochondrial ATP production represents another determinant of donor competence. Reduced mitochondrial energy status impairs cholesterol efflux, whereas improving mitochondrial function can enhance export (90). Lysosomal dysfunction may simultaneously restrict the delivery of free cholesterol from lipid droplets to the plasma membrane.

The effects of death-mode crosstalk on the donor side therefore include:

loss of viable efflux-competent cells;
impaired intracellular cholesterol mobilization;
ATP depletion;
inflammatory repression of cholesterol-handling pathways;
and disruption of membrane transporter localization or activity.

These consequences may occur together, but their relative importance is likely to vary across macrophage subsets and lesion stages.

#### 5.4.3 HDL and extracellular acceptor function

Macrophage cholesterol efflux depends not only on donor-cell transporters but also on the functional quality of apoA-I and HDL. Inflammatory and oxidative remodeling can alter the HDL proteome, oxidize apoA-I, and reduce cholesterol-accepting capacity even when HDL-C concentration remains unchanged (29–32, 45, 96).

Because HDL dysfunction is a shared downstream consequence of plaque inflammation and oxidative stress, it should not be assigned repeatedly to individual death pathways. Pyroptosis, necroptosis, ferroptosis, and secondary necrosis may all contribute to an extracellular environment that impairs HDL function, but pathway-specific attribution requires evidence that manipulation of a defined death executor preserves HDL acceptor capacity independently of generalized changes in inflammation or oxidative stress.

#### 5.4.4 Efferocytosis determines the fate of cellular lipid cargo

Efferocytic competence determines whether intracellular lipid is processed through a controlled clearance pathway or redistributed into the extracellular plaque compartment. Apoptosis followed by efficient efferocytosis preserves membrane integrity and permits lysosomal processing of apoptotic-cell-derived cholesterol, supporting LXR-dependent adaptive cholesterol export (23, 24).

When efferocytosis fails, apoptotic cells undergo secondary necrosis and release cholesterol, oxidized lipids, proteases, and DAMPs that may be re-internalized by neighboring macrophages, thereby increasing foam-cell burden and necrotic-core expansion (7, 21). Primary lytic death produces a similar extracellular redistribution of lipid without the preceding phase of membrane-preserved clearance.

Accordingly, the most relevant distinction for net cholesterol handling may not be the nominal death label alone, but whether membrane integrity is maintained long enough for effective efferocytosis and whether recipient macrophages retain sufficient lysosomal and transporter capacity to dispose of the acquired lipid.

#### 5.4.5 Evidence hierarchy

The evidence supporting the proposed network is not uniform. Relatively direct evidence supports:the local coupling of apoptosis, efferocytosis, lysosomal cholesterol hydrolysis, and adaptive cholesterol efflux (23, 24); the role of lipophagy in mobilizing lipid-droplet cholesterol for ABCA1-dependent efflux (64); the impairment of cellular cholesterol efflux and systemic macrophage RCT by GSDMD-dependent inflammatory signaling (42); and the contribution of macrophage autophagy deficiency, necroptosis, and ferroptosis to experimental plaque progression (50, 55, 56, 71).

By contrast, the following relationships remain indirect or incompletely validated:

direct regulation of ABCA1/ABCG1 by caspase-8;
direct control of macrophage RCT by a specific RIPK1 ubiquitin-chain pattern;
selective suppression of cholesterol transporters by MLKL;
direct impairment of systemic RCT by ferroptosis;
a universal ferroptosis–pyroptosis feed-forward loop;
and a single quantitative ROS or autophagy threshold that determines death-mode selection.

**Figure 1:**
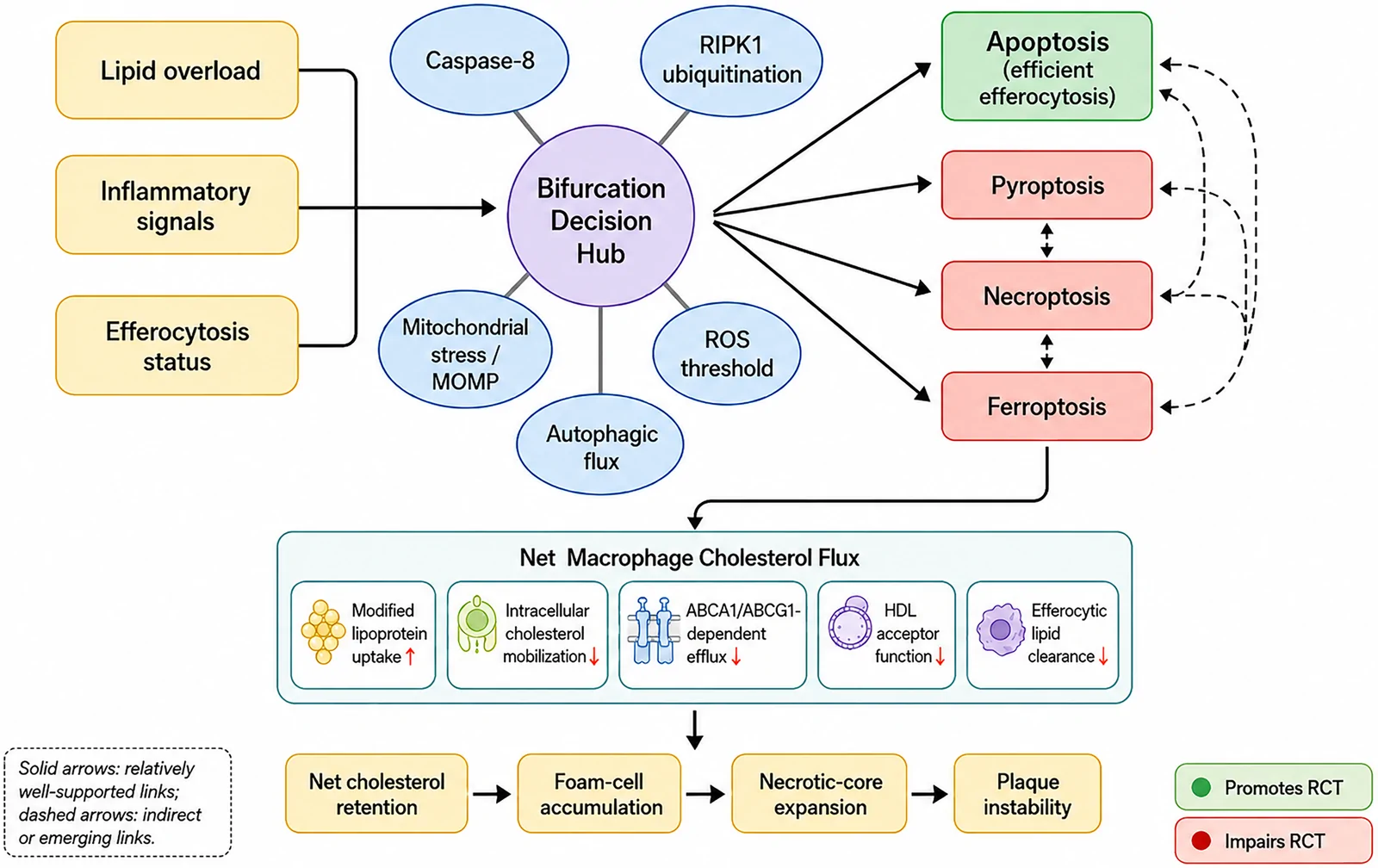
Proposed framework linking macrophage death-mode bifurcation to net cholesterol flux and plaque progression. The schematic presents a multilevel conceptual framework through which macrophage death-mode crosstalk may influence cholesterol homeostasis during atherosclerosis. Environmental inputs, including lipid overload, inflammatory signals, and the functional status of efferocytosis, converge on a central Bifurcation Decision Hub. This hub is regulated by interconnected molecular nodes, including caspase-8 activity, RIPK1 ubiquitination, mitochondrial stress and mitochondrial outer membrane permeabilization (MOMP), oxidative stress and redox state, and autophagic flux. Depending on the intensity, duration, and combination of these signals, macrophages may undergo apoptosis, pyroptosis, necroptosis, or ferroptosis. Apoptosis accompanied by efficient efferocytosis may preserve membrane integrity, promote controlled lipid clearance, and support adaptive cholesterol efflux. By contrast, pyroptosis, necroptosis, and ferroptosis are associated with membrane disruption, inflammatory amplification, oxidative injury, and defective clearance. Crosstalk and switching among these death modalities may further intensify macrophage dysfunction. The downstream effects of this death network are summarized as changes in net macrophage cholesterol flux, including potentially increased modified lipoprotein uptake, impaired intracellular cholesterol mobilization, reduced ABCA1/ABCG1-dependent cholesterol efflux, diminished HDL acceptor function, and defective efferocytic lipid clearance. Together, these alterations favor net cholesterol retention, foam-cell accumulation, necrotic-core expansion, and plaque instability. Solid arrows indicate relatively well-supported relationships, whereas dashed arrows represent indirect, context-dependent, or emerging mechanistic links.

The death-mode bifurcation framework should therefore be viewed as a testable systems-level model rather than a fully established causal network. Future studies should simultaneously measure death-pathway identity, membrane integrity, modified-lipoprotein uptake, intracellular cholesterol mobilization, transporter-dependent efflux, HDL acceptor function, efferocytosis, and systemic RCT within the same experimental system. As shown in Figure 1, this framework integrates these interconnected processes into a systems-level model.

## 6 Conclusions and perspectives

### 6.1 Conclusions

This review integrates current evidence linking macrophage programmed cell death with intracellular cholesterol handling, cholesterol efflux, and reverse cholesterol transport in atherosclerosis. Macrophage cholesterol homeostasis is determined by the balance among modified-lipoprotein uptake, intracellular cholesterol mobilization, transporter-dependent efflux, extracellular acceptor function, and lipid transfer during efferocytosis. Programmed cell death can disturb several of these processes simultaneously, but the strength and directness of the supporting evidence differ substantially among death modalities.

The most direct evidence supports three relationships. First, efficient efferocytosis couples apoptotic-cell clearance to lysosomal cholesterol processing, LXR activation, and adaptive cholesterol export, whereas defective clearance promotes secondary necrosis, lipid redistribution, and plaque instability (7, 23, 24). Second, lipophagy mobilizes lipid-droplet cholesterol for predominantly ABCA1-dependent efflux, provided that lysosomal function, intracellular trafficking, transporter availability, and extracellular acceptor capacity remain intact (64). Third, GSDMD-dependent inflammatory signaling has been shown to impair macrophage cholesterol efflux and the movement of macrophage-derived cholesterol to plasma, liver, and feces, providing the strongest direct connection between a lytic death pathway and systemic macrophage RCT (42).

By comparison, the effects of necroptosis and ferroptosis on cholesterol transport are less directly established. RIPK1/RIPK3/MLKL-dependent necroptosis and iron-dependent ferroptotic injury contribute to macrophage loss, inflammatory amplification, necrotic-core expansion, and experimental plaque progression (50, 51, 55, 56). However, current evidence does not demonstrate that MLKL activation or ferroptotic execution consistently and directly suppresses ABCA1/ABCG1-dependent efflux or systemic RCT. Their metabolic consequences are more appropriately interpreted as arising from loss of viable efflux-competent cells, membrane or organelle injury, extracellular lipid redistribution, inflammatory repression of cholesterol-handling pathways, and deterioration of HDL acceptor function.

Autophagy and lipophagy exert context-dependent effects rather than uniformly protective or harmful actions. Completed autophagic flux and appropriate cargo removal can preserve organelle quality, reduce inflammatory stress, and support intracellular cholesterol mobilization (63, 71). In contrast, ferritinophagy or autophagic GPX4 degradation may increase ferroptotic susceptibility when iron availability rises or antioxidant defenses are depleted (73, 92). The proposed autophagic “optimal window” should therefore be regarded as a testable hypothesis determined by flux completion, cargo selectivity, lysosomal competence, iron and lipid burden, antioxidant capacity, cell lineage, and lesion stage.

The proposed death-mode bifurcation framework places caspase-8 activity, RIPK1 ubiquitination, mitochondrial and lysosomal stress, oxidative status, and autophagic flux within a shared regulatory network. These nodes may influence whether macrophage injury remains compatible with apoptosis and effective clearance or progresses toward inflammatory, oxidative, or lytic death (75, 78). Their relevance to cholesterol metabolism lies primarily in their combined effects on cell viability, membrane integrity, intracellular lipid mobilization, efferocytosis, and extracellular acceptor function rather than in direct regulation of a single cholesterol transporter.

Overall, macrophage death–cholesterol crosstalk is best evaluated at the level of net cholesterol flux rather than isolated changes in ABCA1, ABCG1, or individual death markers. The current framework provides a testable conceptual model, but several proposed connections remain indirect and are derived from separate experimental systems. Future studies should therefore measure death-pathway identity, membrane integrity, lipid uptake, cholesterol mobilization, transporter-dependent efflux, efferocytosis, HDL function, and systemic RCT within the same temporally and cell-specifically resolved models.

### 6.2 Limitations

Several limitations should be considered when interpreting the proposed death-mode bifurcation framework. First, much of the evidence linking macrophage death pathways to cholesterol transport is derived from cultured cells or animal models, and direct validation in human atherosclerotic plaques remains limited. Findings obtained in non-atherosclerotic inflammatory, infectious, or cancer models may not be directly transferable to cholesterol-loaded plaque macrophages.

Second, many studies identify a specific death modality using a restricted number of molecular markers. Because apoptosis, pyroptosis, necroptosis, ferroptosis, and secondary necrosis may coexist or occur sequentially within the same lesion, individual markers may not reliably distinguish pathway identity or demonstrate functional switching between death programs. Future studies should therefore combine morphological assessment, pathway-specific genetic manipulation, functional rescue experiments, and measurements of membrane integrity to define the predominant mode of cell death more rigorously.

Third, relatively few studies have simultaneously measured cell-death identity, macrophage lipid uptake, intracellular cholesterol mobilization, transporter-dependent efflux, efferocytosis, HDL functionality, and whole-body RCT. Consequently, several connections discussed in this review represent mechanistic integration across separate experimental systems rather than directly demonstrated causal pathways.

Fourth, macrophage heterogeneity, lesion stage, sex, metabolic status, comorbidities, and species differences may substantially modify the relationship between cell death and cholesterol flux. The biological effects of the same pathway may also differ among plaque macrophages, vascular smooth muscle cells, endothelial cells, and recruited immune-cell subsets.

Finally, this review provides a conceptual synthesis rather than a quantitative evaluation of effect sizes across studies. The proposed framework should therefore be regarded as a hypothesis-generating and testable working model that requires validation using temporally resolved, cell-specific, and *in vivo* experimental approaches.

### 6.3 Perspectives

Future research should prioritize direct testing of the proposed links between macrophage death identity and net cholesterol flux. Experimental designs should simultaneously assess modified-lipoprotein uptake, intracellular cholesterol esterification and mobilization, ABCA1/ABCG1-dependent efflux, efferocytosis, HDL acceptor function, and cell-death execution within the same biological system. Time-resolved genetic and pharmacological perturbation of caspase-8, RIPK1/RIPK3, gasdermins, MLKL, GPX4, and selective autophagy pathways may help distinguish causal switching mechanisms from parallel responses to common upstream stressors.

The spatiotemporal organization of death-mode crosstalk within plaques also requires further investigation. Single-cell and spatial transcriptomics, proteomics, lipidomics, intravital imaging, and advanced human plaque models may clarify whether different death programs coexist within individual macrophages or occur in distinct cellular subsets and lesion regions. These approaches may also identify how dying cells influence neighboring macrophages through cytokines, oxidized lipids, extracellular vesicles, mitochondrial components, and uncleared cellular debris.

The context-dependent role of autophagy deserves particular attention. Future studies should distinguish autophagy initiation from completed autophagic flux and separately evaluate lipophagy, mitophagy, ferritinophagy, and autophagic GPX4 degradation. Defining how these selective pathways vary with lesion stage, iron burden, lipid composition, lysosomal competence, and antioxidant capacity will be necessary before an “optimal therapeutic window” can be established.

At the translational level, interventions targeting a single death pathway may be insufficient when multiple inflammatory and metabolic processes coexist. Multi-node strategies that combine lipid lowering with modulation of inflammatory cell death, efferocytosis, cholesterol efflux, mitochondrial quality control, or oxidative stress may therefore merit investigation. However, combination approaches should not be assumed to be superior without direct comparative evidence, because simultaneous inhibition of several death pathways may produce compensatory signaling, impair host defense, disrupt physiological cell turnover, or generate unanticipated effects on plaque composition.

Macrophage-targeted delivery systems may improve therapeutic selectivity and reduce systemic adverse effects. Candidate interventions directed at caspase-8, RIPK1 ubiquitination, inflammasome–gasdermin signaling, GPX4 preservation, autophagic flux, or ABCA1 stability should be evaluated in lesion-stage-specific and cell-type-specific models before clinical translation. Ultimately, integrating molecular markers of macrophage death with imaging findings, lipid parameters, inflammatory biomarkers, and functional measures of HDL may support more precise risk stratification. Such studies may help determine whether targeting macrophage death–cholesterol crosstalk can provide additional cardiovascular benefit beyond conventional LDL-C lowering.
